# Oral health status in children with chronic kidney disease, kidney transplantation, and nephrotic syndrome: a cross-sectional study

**DOI:** 10.1007/s00467-025-06698-1

**Published:** 2025-02-04

**Authors:** Anna Beyer, Fabian Ebach, Heiko Reutter, Katja Sauerstein, Alina Christine Hilger, Tobias Krickau, Anja Tzschoppe, Joachim Woelfe, Matthias Galiano, Jan Thomas Schaefer

**Affiliations:** 1 Friedrich-Alexander Erlangen-Universität, Schloßplatz 4, 91054 Erlangen, Germany; 2 Department of Neonatology and Pediatric Intensive, Care University Hospital Bonn, Bonn, Germany; 3 Department of Pediatrics and Adolescent Medicine, Division of Neonatology and Pediatric Intensive Care, University Hospital Erlangen, Erlangen, Germany; 4 Department of Pediatrics and Adolescent Medicine, Department of Pediatric Nephrology, University Hospital Erlangen, Erlangen, Germany

**Keywords:** Chronic kidney disease, Kidney transplant recipients, Nephrotic syndrome, DDE, OHI-S, DMFT

## Abstract

**Introduction:**

Chronic kidney disease (CKD) has been previously associated with a decline in oral health. This study aimed to examine the oral health of children with CKD, nephrotic syndrome (NS), and children that received kidney transplantation (KTR).

**Methods:**

A case–control study was conducted involving children with CKD stages 1–3, children with CKD stages 4–5, pediatric kidney transplant recipients, and children with NS. Developmental Defects of Enamel (DDE) were evaluated using the DDE Index, while dental caries was assessed with the Decayed Missing Filled Teeth Index (DMFT). Plaque and debris were measured utilizing the Simplified Oral Hygiene Index (OHI-S), which includes the two subindices Simplified Calculus Index (CI-S) and Simplified Debris Index (DI-S).

**Results:**

Children with CKD 1–3, CKD 4–5, and KTR presented with significantly higher DI-S and CI-S scores and significantly more DDE. There was no difference in the DMFT score in children with CKD 4–5 and KTR. For children with CKD 1–3, a significantly lower DMFT score was observed compared to the control group. Children with NS did not show any differences for DI-S, CI-S, DMFT, and DDE compared to healthy peers.

**Conclusion:**

Oral health status is not affected in children with NS. Children with CKD 1–3, CKD 4–5, and KTR have more plaque, debris, and DDE and should be surveyed regularly by their dentists.

**Graphical Abstract:**

**Figure:**
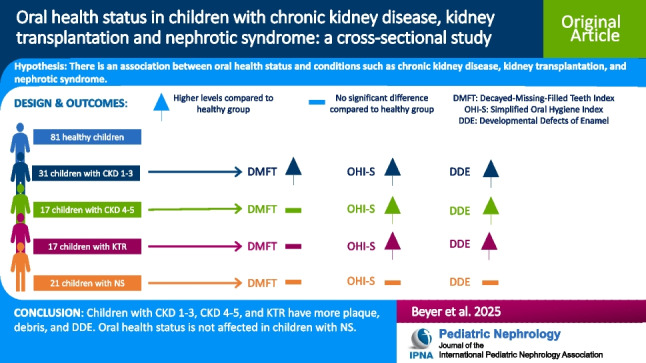
A higher resolution version of the Graphical abstract is available as Supplementary information

**Supplementary Information:**

The online version contains supplementary material available at 10.1007/s00467-025-06698-1.

## Introduction

Chronic kidney disease (CKD) represents a global issue in pediatric health care. Approximately 15 to 74.7 per million children are affected by (CKD) [1]. CKD can be classified into five stages based on the glomerular filtration rate [2]. In CKD V, also known as kidney failure, long-term survival requires dialysis or kidney transplantation [3]. Kidney transplantation (KTR) is the preferred treatment for children with kidney failure [4]. Nephrotic syndrome (NS) in children is a rare disease that leads to proteinuria, hypoalbuminemia, edema, and hyperlipidemia and affects about 1 in 20,000 children [5, 6]. Treatment unresponsiveness often ends up in kidney failure.

CKD in children is associated with comorbidities such as failure to thrive, secondary hyperparathyroidism, hyperphosphatemia, metabolic acidosis, and renal anemia [5]. Oral symptoms have been associated with CKD since 1851 [7]. These include lower dental caries scores, reduced salivary flow rate, high prevalence of calculus, debris, gingivitis, enamel hypoplasia, and a higher prevalence of developmental defects of enamel compared to their healthy peers [8, 9]. NS can be associated with severe gingivitis, increased developmental defects in enamel, and increased accumulation of plaque and debris. However, it remains uncertain whether it is associated with caries [10, 11].

The impact of CKD, KTR, and NS on the oral health of children remains unclear. Therefore, this study aimed to investigate possible differences in oral health status in children with CKD, KTR and NS under the assumption that no significant difference exists in these groups compared to their healthy peers.

## Material and methods

### Ethical approval

This study was performed in line with the principles of the Declaration of Helsinki. Approval was granted by the Ethics Committee of Friedrich-Alexander Universität Erlangen-Nürnberg (06.2023/23–214-B). Before examining the patients, informed consent was obtained from the parents or caregivers.

### Study population

The oral health status of 167 children between four and seventeen years of age who had regular check-ups at the Center for Pediatric Nephrology and Dialysis at the University of Erlangen/Nuremberg was examined in this study. The children were divided into five groups: CKD stage 1–3, CKD stage 4–5, KTR, NS, and a group of healthy control peers. The children in the healthy control group were recruited from the same center as the children with CKD, KTR and NS during an outpatient visit. However, they did not have NS, KTR or any form of CKD. The glomerular filtration rate was determined according to the revised Schwartz Equation from 2009 as follows: eGFR (ml/min × 1.73 m^2^) = 0.413 × Height (cm)/Serum creatinine (mg/dl) [12]. The KDIGO nomenclature was used to classify children with CKD into the respective stages: Stage 1 with an eGFR ≥ 90 ml/min/1.73 m^2^, stage 2 with an eGFR between 60 and 89 ml/min/1.73 m^2^, stage 3 with an eGFR between 30 and 59 ml/min/1.73 m^2^, stage 4 with an eGFR between 15 and 29 ml/min/1.73 m^2^, and stage 5 with an eGFR under 15 ml/min/1.73 m^2^. [2]

The CKD groups were clustered into stage 1–3 and 4–5 due to CKD being mostly asymptomatic in stages 1–3. In stages 4 and 5, children usually show symptoms of CKD [13].

### Clinical data

For each child, sex, date of birth, age, height, weight, serum creatinine level, and disease status were recorded.

### Examination

The examination was performed during a regular visit at the Center for Pediatric Nephrology and Dialysis at the University of Erlangen/Nuremberg. Each examination started with an informative conversation about the study with a parent or caregiver and the child. An information sheet was provided both to parents and children, explaining the background and purpose of the study. Two different information sheets were used: one for children aged 8–12, and another one for children aged 13–17. Such information sheets for different age groups are required by the ethics committee of Friedrich-Alexander Universität Erlangen-Nürnberg. In addition, the process and the study was explained in detail using age-appropriate language. Pre-examination informed consent was given to the parent or caregiver. The examination had no impact on other treatments in the clinic. An oral mirror and dental loupes with light were used for every intraoral examination. The examination was performed consistently by the same person in an identical environment. The examiner was an advanced dental student with prior clinical practice experience at the university. The examination took approximately two to three minutes.

No blood had to be drawn for study purposes. However, due to other routine examinations conducted on the same day as the dental examination, study-relevant blood values were available. For most children, the blood values were taken on the same day. The maximum time span for blood collection was within one month before or after examination. Weight and height were also determined on the day of the dental examination during normal check-up.

### Indices

#### Decayed missing filled teeth index (DMFT)

The DMFT index determines the prevalence of dental caries. Therefore, a sum is formed by adding the number of decayed teeth due to caries, missing teeth due to caries, and filled teeth. The index is written in lowercase in the primary dentition (dmft) and in uppercase in the permanent dentition (DMFT). In mixed dentition, only permanent teeth are considered (DMFT) [14]. In this paper, the DMFT index is not considered separately for primary and permanent dentition.

#### Simplified oral hygiene index (OHI-S)

The OHI-S index is used to determine dental calculus and debris. It has two subindices, the Calculus Index (CI-S) and the Debris Index (DI-S). Overall, six tooth surfaces are examined, including four posterior and two anterior teeth: 16 vestibular, 26 vestibular, 11 vestibular, 36 lingual, 46 lingual and 11 lingual. In total, there are four stages: stage 0 no calculus or debris; stage 1 calculus or debris covers the gingival third of the tooth; stage 2 calculus or debris covers 1/3 to 2/3 of the gingival tooth surface; stage 3 debris or calculus covers more than 2/3 of the tooth surface. Each of the six tooth surfaces is assigned a stage for the CI-S index. These six stages are added and divided by six, meaning the number can range from zero to three. Afterward, the same is done for the DI-S Index. Lastly, the CI-S and DI-S results are added to arrive at the OHI-S Index, meaning the number can range from zero to six [15, 16].

#### Developmental defects of enamel (DDE)

The FDI provides this index [17]. In this study, children could either have no developmental defects of enamel, diffuse developmental defects of enamel, or demarcated developmental defects of enamel.

### Statistical analysis

Statistical analysis was performed using R version 4.3.0 [18]. As all the dental indices are non-normal distributed ordinal data, we used individual Mann–Whitney-Wilcoxon tests to compare each disease group to the control group. The statistical levels are therefore to be interpreted in relation to the control group. For each index, given p-values were corrected for the number of comparisons using the Bonferroni method. An alpha value of *p* < 0.05 was considered as statistically significant.

## Results

As shown in Table 1, 167 children were included in this study. Among them, 81 were healthy controls, 31 were diagnosed with CKD 1–3, 17 with CKD 4–5, 17 had previous KTR, and 21 were affected by NS. The median age in the healthy group was 9 years; in the CKD 1–3 group it was 11 years; in the CKD 4–5 group it was 10 years; in the KTR, it was 12 years; and in the NS group, it was 8 years.

**Table 1:** Age of control group, different stages of chronic kidney disease (CKD), kidney transplant recipients (KTR) and nephrotic syndrome (NS)

	Control Median (IQR) *n* = 81	CKD 1–3 Median (IQR) *n* = 31	CKD 4–5 Median (IQR) *n* = 17	KTR Median (IQR) *n* = 17	NS Median (IQR) *n* = 21
Age	9[7;13]	11 [8;15]	10[5.75; 14.25]	12 [10;14]	8 [7;12]

^*^p < 0.05, **p < 0.01, ***p < 0.001, ****p < 0.0001

n, number; IQR, interquartile range; CKD, chronic kidney disease; KTR, kidney transplant recipients; NS, nephrotic syndrome

In Table 2 the median DMFT score is displayed. The healthy group had a median DMFT score of 1, the children with CKD 1–3 and CKD 4–5 had a median DMFT score of 0, the KTR had a median DMFT score of 2, and the children with NS a median score of 2. There was no significant difference in caries status between the CKD 4–5 (*p* = 0.312), KTR (*p* = 0.057) and NS (*p* = 0.312) groups compared with the healthy controls (p > 0.05). However, the DMFT score differed significantly between the CKD 1–3 and the healthy group (*p* = 0.019).

**Table 2:** DMFT and simplified debris, calculus and oral hygiene indices in a control group, different stages of chronic kidney disease (CKD), kidney transplant recipients (KTR) and nephrotic syndrome (NS)

	Control *n* = 81 Median (IQR)	CKD 1–3 *n* = 31 Median (IQR)	CKD 4–5 *n* = 17 Median (IQR)	KTR *n* = 17 Median (IQR)	NS *n* = 21 Median (IQR)
DMFT	1 [0;2]	0 [0;0.5]*	0 [0;1]	2 [1;4]	2 [0;3]
DI-S	0.33 [0;0,5]	0.83 [0.5;1]****	1 [1;1.5]****	0.67 [0.33;1]**	0.5 [0.33;0.67]
CI-S	0.17 [0;0.5]	0.67 [0.5;1]****	1 [0.5;2]****	0.83 [0.29;1]***	0.33 [0;0.5]
OHI-S	0.5 [0;1]	1.5 [1;2]****	2 [1.5;3.33]****	1.33 [0.83;2]***	0.83 [0.33;1.17]

^*^p < 0.05, **p < 0.01, ***p < 0.001, ****p < 0.0001

n, number; IQR, interquartile range; CKD, chronic kidney disease; KTR, kidney transplant recipients; NS, nephrotic syndrome; DMFT, decayed-missing-filled; DI-S, simplified debris index; CI-S, simplified calculus index; OHI-S, simplified oral hygiene index

In addition, Table 2 shows the groups' CI-S, DI-S, and OHI-S scores. Higher median CI-S scores could be observed in the CKD 1–3 group (CI-S = 0.67), in the CKD 4–5 group (CI-S = 1), in the KTR group (CI-S = 0.83), and in the NS group (CI-S = 0.33) compared to the control group (CI-S = 0.17). There was a significant association in the median CI-S score between CKD 1–3 (*p* < 0.0001), CKD 4–5 (*p* < 0.0001), and KTR (p < 0.001) and the control group, in contrast to the NS (*p* = 0.332) group, where no significant correlation could be viewed for the median CI-S score compared to the healthy group.

The group of healthy control peers had a median DI-S score of 0.33. In comparison, children with CKD 1–3, CKD 4–5, KTR, and NS all expressed a higher median DI-S score than the healthy group (CKD 1–3: 0.83; CKD 4–5: 1; KTR: 0.67; NS: 0.5). This was significant for the CKD 1–3 (*p* < 0.0001), CKD 4–5 (*p* < 0.0001) and KTR (*p* < 0.01) groups, but not for the NS (*p* = 0.072) group. Subsequently, the OHI-S score, which is composed of the DI-S score and the CI-S score, was higher compared to the control group in all four groups. In comparison to their healthy peers, the OHI-S score was significant for the CKD 1–3 (*p* < 0.0001), CKD 4–5 (*p* < 0.0001), and KTR (*p* < 0.001) groups but not for the NS (*p* = 0.129) group.

In this study, 72% of the healthy children, 45% of those with CKD 1–3, 29% of patients with CKD 4–5, 24% with KTR, and 71% of those with NS expressed no DDE. Twenty-five percent of the healthy children, 35% of the CKD 1–3 group, 41% of the CKD 4–5 group, 29% of the KTR group, and 14% of the NS group exhibited demarcated DDE. Diffuse DDE were observed in 4% of the children in the healthy group, 19% in the CKD 1–3 group, 24% in the CKD 4–5 group, 47% in the KTR group, and 14% in the NS group. A statistically significant relationship was found between these observations for the CKD 1–3 (*p* < 0.01), CKD 4–5 (*p* < 0.01), and KTR (*p* < 0.0001) group. No statistical significance was observed for the children with NS (*p* = 0.781).

## Discussion

This study aimed to examine the oral health status of children with CKD, previous KTR, or NS and compare it to a group of healthy control peers. Several studies have recently highlighted the influence of CKD and kidney transplantation on oral health status [9, 19, 20]. This study demonstrates that CKD 1–5 and KTR significantly influence debris and calculus accumulation, as well as the prevalence of DDE. In contrast, NS did not have an impact on debris and calculus accumulation or DDE. Notably, only CKD 1–3 was associated with an increased number of carious lesions.

DDE occur when ameloblasts are damaged or their metabolic activity is disturbed during enamel formation. These defects are irreversible and can be diffuse or demarcated [14]. The disrupted metabolic activity in CKD can be attributed to hypocalcemia [5], hyperphosphatemia [5], decreased serum levels of 1,25-dihydroxycholecalciferol [21], and fluorosis [20, 22]. Just as several studies have presented that there is a higher prevalence of DDE in children with CKD [8, 23, 24] the present study confirmed these findings highlighting the importance of disrupted metabolic activity in children with CKD [5, 14, 21, 22]. The highest levels of DDE in our study were observed in KTR. This might be because children with KTR may have experienced various stages of CKD earlier in life prior to KTR, including the critical period for tooth development [25]. Studies have indicated that children with NS exhibit more developmental defects of enamel than those without the condition [10, 11]. Nevertheless, our study did not support this finding. We found children with NS did not show any differences in DDE compared to healthy peers. However, in our study we did not divide children with NS into subgroups of different clinical courses and etiologies. Children with NS show changes in calcium, vitamin D, and phosphate metabolism [26, 27] which could be a reason that children with NS have more DDE.

Debris refers to a structured, yellowish dental plaque composed of microorganisms embedded within a protein matrix and a polysaccharide-based matrix. Failure to remove plaque through regular tooth brushing and interdental cleaning leads to calculus formation. Calculus is mineralized debris [28]. Saliva in children with CKD has a higher pH level and contains elevated amounts of urea and phosphorus, which then combine to form calcium-phosphorus and calcium oxalate [20]. This in in accordance with earlier studies, which indicated that children with CKD have more calculus and debris accumulation than healthy children [8, 9]; our study confirmed this observation. The higher debris and plaque accumulation is due to the higher pH level of the saliva in children with CKD and KTR [20]. For children with NS, the prevalence was higher in debris and calculus in other studies [10, 11]. Our study did not show differences compared to the healthy peers. The higher levels in debris and calculus in other studies could be due to a raised pH level in the saliva of this group [29].

Dental caries is a process caused by cariogenic bacteria, particularly Streptococcus mutans, which form a biofilm on the tooth surface [30]. These bacteria metabolize carbohydrates, producing acids that gradually demineralize the tooth surface [30]. The process is dynamic, involving cycles of demineralization and remineralization, with demineralization predominating over time [30]. Children with CKD often experience xerostomia [31], reduced saliva flow [8]. Furthermore, it has been assumed that they may have a higher intake of cariogenic snacks compared to their healthy peers [32]. Although these factors would typically indicate a higher likelihood of developing caries, this could not be observed in recent studies.

Numerous studies have investigated the occurrence of dental caries in children with CKD, whereby most have found that the DMFT score is lower in these children compared to a healthy control group [8, 9, 24, 33]. Additionally, two studies examined the oral health of children undergoing hemodialysis and reported differing results. One study found that the incidence of dental caries was higher in these children [34], while the other study found it to be lower [35]. Our study revealed that children with CKD 1–3 and CKD 4–5 had fewer dental caries than the control group, though this difference was not statistically significant for the children with CKD 4–5. Conversely, children who underwent KTR had more dental caries in our study than the control group, although this was non-significant. This was not the case in another recent study where the children with kidney transplantation had a lower DMFT score than the control group [23]. The lower DMFT score in children with CKD could be attributed to a more alkaline oral pH of the saliva and a reduced presence of Streptococcus mutans [36, 37]. The higher pH level of the saliva worsens the environment for the cariogenic bacteria, leading to a slower formation of dental caries [20]. The higher DMFT score (non-significant) in our research of children with KTR compared to their healthy peers could be due to increased Streptococcus mutans levels following transplantation [36], which could lead to a rise in the primary cariogenic bacteria in the biofilm, which could be investigated in future studies. In addition, teeth with DDE are more susceptible to caries due to structural irregularities on the tooth surface created by the DDE. These structural irregularities lead to calculus and debris accumulation, which are hard to remove because they are hard to reach with a toothbrush. This fosters an environment that contributes to the development of caries [38]. As children with CKD 1–5 and KTR experience more DDE in our study this could contribute to an increased accumulation of calculus and debris, ultimately leading to a higher incidence of carious lesions. However, this could not be proven in our study and more data should be obtained to elucidate this aspect. The carious score in NS was higher [10] but lower [11] in another study. Our study showed non-significant higher dental carious scores. A recent study found that children with idiopathic NS had Streptococcus mutans levels comparable to their healthy peers. This suggests that children with NS are not expected to have more carious lesions than their healthy peers [11].

Our study has several limitations. The groups were small, only representing a fraction of the patients with CKD 1–3, CKD 4–5, those who had undergone kidney transplantation and NS. Group combination was performed as five groups would have led to a very small sample size in each group. Classification in these CKD groups was based on clinical features, as patients with CKD 1–3 classically do not have severe clinical problems, whereas patients with CKD 4–5 suffer from different comorbidities [13]. Therefore, it appeared likely that combining these groups could be sufficient to demonstrate statistical differences. Moreover, the children with idiopathic NS were not divided into subgroups of different clinical courses and etiologies. The misbalance of the study groups may affect the reliability of statistical results. Additionally, dental caries was examined visually without bitewing radiographs or an orthopantomogram. Due to this, interproximal caries could have been overlooked. Furthermore, the study did not assess the number or severity of developmental defects of enamel. Another limitation is that no disclosing agents or instruments were used for dental examination. Moreover, this study did not evaluate other comorbidities aside from kidney disease, which may potentially confound the results. For feasibility reasons, inter- and intra-rater reliability scores were not assessed, which is another limitation of this study.

On the other hand, strengths of this study included the use of reproducible indices and the relatively large patient numbers in each of the study groups. This ensures consistent examinations and the possibility of comparing it to other studies. Additionally, the control group and the group of kidney transplant recipients were larger than in other studies.

In conclusion, children with CKD and KTR have dental disease and oral health problems in a considerable number of cases highlighting the structural defects of enamel in CKD. Furthermore, debris, plaque and DDE are also increased. However, NS does not have a significant impact on children’s oral health in our study.

## Supplementary Information

Below is the link to the electronic supplementary material.

Graphical abstract (PPTX 78 KB)

## Data Availability

The data supporting this study are not publicly available due to privacy concerns regarding sensitive information about the children involved.

## References

[1] Warady BA, Chadha V (2007) Chronic kidney disease in children: the global perspective. Pediatr Nephrol 22:1999–2009. 10.1007/s00467-006-0410-1 17310363 10.1007/s00467-006-0410-1 PMC2064944

[2] Kidney Disease: Improving Global Outcomes (KDIGO) CKD Work Group (2024) KDIGO 2024 Clinical Practice Guideline for the Evaluation and Management of Chronic Kidney Disease. Kidney Int 105:S117–S314. 10.1016/j.kint.2023.10.018 38490803 10.1016/j.kint.2023.10.018

[3] Agarwal R (2015) Defining end-stage renal disease in clinical trials: a framework for adjudication. Nephrol Dial Transplant 31:864–867. 10.1093/ndt/gfv289 26264780 10.1093/ndt/gfv289

[4] van Heurn E, de Vries EE (2009) Kidney transplantation and donation in children. Pediatr Surgery Int 25:385–393. 10.1007/s00383-009-2350-x 10.1007/s00383-009-2350-x 19330513

[5] Piper W (2013) Innere Medizin [Internal medicine]. Springer, Heidelberg, pp 284–288

[6] Banh THM, Hussain-Shamsy N, Patel V, Vasilevska-Ristovska J, Borges K, Sibbald C, Lipszyc D, Brooke J, Geary D, Langlois V, Reddon M, Pearl R, Levin L, Piekut M, Licht CPB, Radhakrishnan S, Aitken-Menezes K, Harvey E, Hebert D, Piscione TD, Parekh RS (2016) Ethnic Differences in Incidence and Outcomes of Childhood Nephrotic Syndrome. Clin J Am Soc Nephrol 11:1760–1768. 10.2215/cjn.00380116 27445165 10.2215/CJN.00380116 PMC5053779

[7] Franz V, Fahr T (1851) Die Brightsche Nierenkrankheit [Bright’s kidney disease]. Kessinger Publishing, Braunschweig

[8] Andaloro C, Sessa C, Bua N, Mantia I (2018) Chronic kidney disease in children: Assessment of oral health status. Dent Med Probl 55:23–28. 10.17219/dmp/81747 30152631 10.17219/dmp/81747

[9] Sezer B, Kaya R, KodamanDokumacıgil N, Sıddıkoğlu D, Güven S, Yıldız N, Alpay H, Kargül B (2023) Assessment of the oral health status of children with chronic kidney disease. Pediatr Nephrol 38:269–277. 10.1007/s00467-022-05590-6 35499576 10.1007/s00467-022-05590-6

[10] Luong HM, Nguyen TT, Tran HT, Tran PT, Nguyen PN, Nguyen HT, Nguyen DM, Duc HTT, Tong SM (2021) Oro-Dental Health and Primary Nephrotic Syndrome among Vietnamese Children. Children (Basel) 8:494. 10.3390/children8060494 34200617 10.3390/children8060494 PMC8229337

[11] Kaczmarek U, Wrzyszcz-Kowalczyk A, Jankowska K, Prościak K, Mysiak-Dębska M, Przywitowska I, Makulska I (2020) Oral health conditions in children with idiopathic nephrotic syndrome: a cross-sectional study. BMC Oral Health 20:213. 10.1186/s12903-020-01197-1 32727436 10.1186/s12903-020-01197-1 PMC7391815

[12] Schwartz GJ, Muñoz A, Schneider MF, Mak RH, Kaskel F, Warady BA, Furth SL (2009) New equations to estimate GFR in children with CKD. J Am Soc Nephrol 20:629–637. 10.1681/asn.2008030287 19158356 10.1681/ASN.2008030287 PMC2653687

[13] Vaidya SR, Aeddula NR (2024) Chronic Kidney Disease. In: StatPearls [Internet]. Treasure Island (FL): StatPearls Publishing. https://www.ncbi.nlm.nih.gov/books/NBK535404/. Accessed December 16 2024

[14] Hellwig E, Schäfer E, Klimek J, Attin T (2023) Einführung in die Zahnerhaltung [Introduction to Conservative Dentistry]. Deutscher Zahnärzteverlag, Köln, p 70

[15] Siegwart P (1978) Prophylaxe [Prophylaxis]. Die Quintessenz, Berlin, p 348

[16] Ehrenfeld M, Gängler P, Hoffmann T, Schwenzer N, Willershausen B (2010) Konservierende Zahnheilkunde und Parodontologie [Conservative Dentistry and Periodontology]. Thieme, Stuttgart, pp 251–252

[17] (1992) A review of the developmental defects of enamel index (DDE Index): Commission on Oral Health, Research & Epidemiology. Report of an FDI Working Group. Int Dent J 42:411–426 1286924

[18] R Foundation for Statistical Computing (2023) A Language and Environment for Statistical Computing. https://www.R-project.org. Accessed 25.04.2024

[19] Subramaniam P, Gupta M, Mehta A (2012) Oral health status in children with renal disorders. J Clin Pediatr Dent 37:89–93. 10.17796/jcpd.37.1.7l913347q0232v01 23342573 10.17796/jcpd.37.1.7l913347q0232v01

[20] Velan E, Sheller B (2021) Oral health in children with chronic kidney disease. Pediatr Nephrol 36:3067–3075. 10.1007/s00467-020-04913-9 33528633 10.1007/s00467-020-04913-9

[21] Galassi A, Fasulo EM, Ciceri P, Casazza R, Bonelli F, Zierold C, Calleri M, Blocki FA, Palmieri MA, Mastronardo C, Cozzolino MG (2022) 1,25-dihydroxyvitamin D as Predictor of Renal Worsening Function in Chronic Kidney Disease. Results From the PASCaL-1,25D Study. Front Med (Lausanne) 9:840801. 10.3389/fmed.2022.840801 35308556 10.3389/fmed.2022.840801 PMC8924653

[22] Fernando W, Nanayakkara N, Gunarathne L, Chandrajith R (2020) Serum and urine fluoride levels in populations of high environmental fluoride exposure with endemic CKDu: a case-control study from Sri Lanka. Environ Geochem Health 42:1497–1504. 10.1007/s10653-019-00444-x 31641912 10.1007/s10653-019-00444-x

[23] Sezer B, KodamanDokumacigil N, Kaya R, Guven S, Turkkan ON, Cicek N, Alpay H, Kargul B (2023) Association between serum biomarkers and oral health status in children with chronic kidney disease: A cross-sectional study. Clin Oral Investig 27:3731–3740. 10.1007/s00784-023-04989-1 37014503 10.1007/s00784-023-04989-1

[24] Limeira FIR, Yamauti M, Moreira AN, Galdino TM, de Magalhães CS, Abreu LG (2019) Dental caries and developmental defects of enamel in individuals with chronic kidney disease: Systematic review and meta-analysis. Oral Dis 25:1446–1464. 10.1111/odi.12993 30338628 10.1111/odi.12993

[25] Nota A, Palumbo L, Pantaleo G, Gherlone EF, Tecco S (2020) Developmental Enamel Defects (DDE) and Their Association with Oral Health, Preventive Procedures, and Children’s Psychosocial Attitudes towards Home Oral Hygiene: A Cross-Sectional Study. Int J Environ Res Public Health 17:4025. 10.3390/ijerph17114025 32516977 10.3390/ijerph17114025 PMC7311990

[26] Feinstein S, Becker-Cohen R, Rinat C, Frishberg Y (2006) Hyperphosphatemia is prevalent among children with nephrotic syndrome and normal renal function. Pediatr Nephrol 21:1406–1412. 10.1007/s00467-006-0195-2 16897004 10.1007/s00467-006-0195-2

[27] Esmaeeili M, Azarfar A, Hoseinalizadeh S (2015) Calcium and Vitamin D Metabolism in Pediatric Nephrotic Syndrome; An Update on the Existing Literature. Int J Pediatr 3:103–109. 10.22038/ijp.2015.3932

[28] Thomas W (2024) Memorix Zahnmedizin [Memorix Dentistry]. Thieme, Stuttgart, pp 43–45

[29] Kaczmarek U, Wrzyszcz-Kowalczyk A, Jankowska K, Prościak K, Mysiak-Dębska M, Przywitowska I, Makulska I (2021) Selected salivary parameters in children with idiopathic nephrotic syndrome: a preliminary study. BMC Oral Health 21:17. 10.1186/s12903-020-01375-1 33413282 10.1186/s12903-020-01375-1 PMC7791758

[30] Krzyściak W, Jurczak A, Kościelniak D, Bystrowska B, Skalniak A (2014) The virulence of Streptococcus mutans and the ability to form biofilms. Eur J Clin Microbiol Infect Dis 33:499–515. 10.1007/s10096-013-1993-7 24154653 10.1007/s10096-013-1993-7 PMC3953549

[31] Martins C, Siqueira WL, Guimarães Primo LS (2008) Oral and salivary flow characteristics of a group of Brazilian children and adolescents with chronic renal failure. Pediatr Nephrol 23:619–624. 10.1007/s00467-007-0718-5 18228044 10.1007/s00467-007-0718-5

[32] Lindeback R, Abdo R, Schnabel L, Le Jambre R, Kennedy SE, Katz T, Ooi CY, Lambert K (2023) Does the Nutritional Intake and Diet Quality of Children With Chronic Kidney Disease Differ From Healthy Controls? A Comprehensive Evaluation. J Ren Nutr 34:283–293. 10.1053/j.jrn.2023.12.002 38128854 10.1053/j.jrn.2023.12.002

[33] Menezes CR, Pereira AL, Ribeiro CC, Chaves CO, Guerra RN, Thomaz EB, Monteiro-Neto V, Alves CM (2019) Is there association between chronic kidney disease and dental caries? A case-controlled study. Med Oral Patol Oral Cir Bucal 24:e211–e216. 10.4317/medoral.22737 30818314 10.4317/medoral.22737 PMC6441594

[34] Pakpour AH, Kumar S, Fridlund B, Zimmer S (2015) A case-control study on oral health-related quality of life in kidney disease patients undergoing haemodialysis. Clin Oral Investig 19:1235–1243. 10.1007/s00784-014-1355-6 25395347 10.1007/s00784-014-1355-6

[35] Andrade MR, Salazar SL, de Sá LF, Portela M, Ferreira-Pereira A, Soares RM, Leão AT, Primo LG (2015) Role of saliva in the caries experience and calculus formation of young patients undergoing hemodialysis. Clin Oral Investig 19:1973–1980. 10.1007/s00784-015-1441-4 25786587 10.1007/s00784-015-1441-4

[36] Al Nowaiser A, Lucas VS, Wilson M, Roberts GJ, Trompeter RS (2004) Oral health and caries related microflora in children during the first three months following renal transplantation. Int J Pediatr Dentistry 14:118–126. 10.1111/j.1365-263X.2004.00534.x 10.1111/j.1365-263x.2004.00534.x 15005700

[37] Ertuğrul F, Elbek-Cubukçu C, Sabah E, Mir S (2003) The oral health status of children undergoing hemodialysis treatment. Turk J Pediatr 45:108–113 12921296

[38] Manton DJ, Vieira AR (2023) Chapter 4: Development defects of enamel and dentine and coronal caries. Monogr Oral Sci 31:37–49. 10.1159/000530556 37364549 10.1159/000530556

