# Supplementary material for: Oral health status in children with chronic kidney disease, kidney transplantation, and nephrotic syndrome: a cross-sectional study
Source: Pediatr Nephrol. 2025 Feb 4;40(7):2287–93. doi: 10.1007/s00467-025-06698-1 (PMC12117002; doi:10.1007/s00467-025-06698-1)
Supplement: Supplementary file 1 — Graphical abstract (PPTX 78 KB) [file 467_2025_6698_MOESM1_ESM.pptx]

## Slide 1
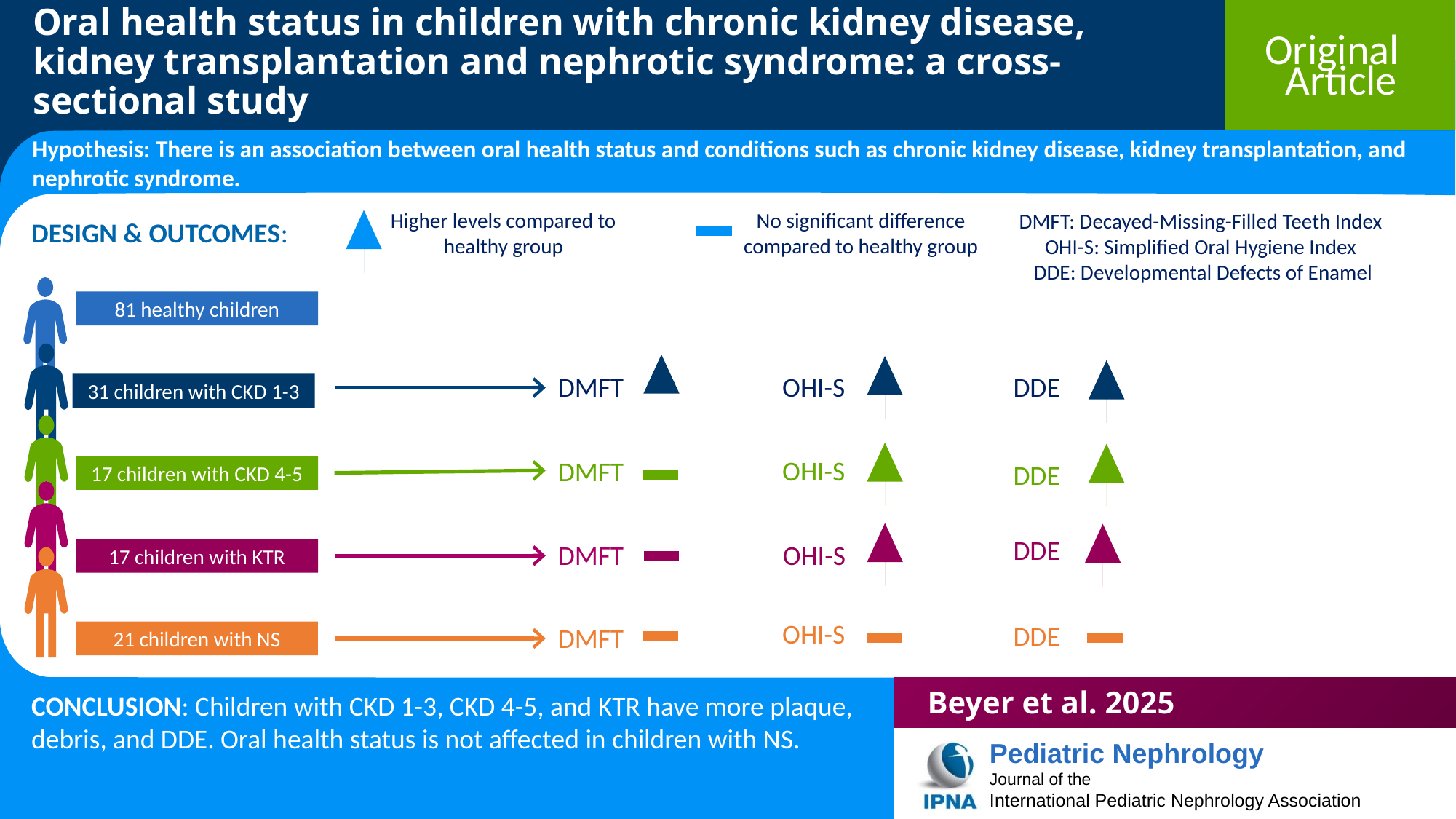

Oral health status in children with chronic kidney disease, kidney transplantation and nephrotic syndrome: a cross-sectional study
Hypothesis: There is an association between oral health status and conditions such as chronic kidney disease, kidney transplantation, and nephrotic syndrome.
No significant difference compared to healthy group
Higher levels compared to healthy group
DMFT: Decayed-Missing-Filled Teeth Index
OHI-S: Simplified Oral Hygiene Index
DDE: Developmental Defects of Enamel
DESIGN & OUTCOMES:
81 healthy children
DMFT
OHI-S
DDE
31 children with CKD 1-3
OHI-S
DMFT
DDE
17 children with CKD 4-5
DDE
DMFT
OHI-S
17 children with KTR
OHI-S
DDE
DMFT
21 children with NS
Beyer et al. 2025
CONCLUSION: Children with CKD 1-3, CKD 4-5, and KTR have more plaque, debris, and DDE. Oral health status is not affected in children with NS.
